# Recent Approaches to Wound Treatment—Second Edition

**DOI:** 10.3390/ijms25105388

**Published:** 2024-05-15

**Authors:** Cinzia Pagano, César Antonio Viseras Iborra, Luana Perioli

**Affiliations:** 1 Department of Pharmaceutical Sciences, University of Perugia, 06100 Perugia, Italy; luana.perioli@unipg.it; 2 Department of Pharmacy and Pharmaceutical Technology, Faculty of Pharmacy, University of Granada, 18071 Granada, Spain; cviseras@ugr.es

Wound healing is a complex process involving a number of mechanisms. Skin repair can be influenced by many factors that may not be directly related to the wound itself; thus, choosing the most appropriate treatment is often difficult.

The aim of this Special Issue is to offer an overview of the possible approaches to wound treatment while considering some of the factors that can affect the healing process.

Many studies have demonstrated that this process can be delayed in the presence of comorbid conditions [1].

Park et al. [2] investigated the role of rheumatoid arthritis (RA), an autoimmune disorder, in delaying wound healing. More specifically, they studied the suitability of using the bioactive peptide AESIS-1, known for its anti-rheumatoid arthritis properties, as an active ingredient for wound-healing promotion. In an in vivo diabetic wound-healing model (BALB/c-nude mice), they observed a stimulation of wound closure when very low doses of AESIS-1 (0.5 µg/wound) were used, in comparison to a control group, represented by wounds treated with PBS. During a period of 12 days, in fact, complete wound closure was observed in the treatment group vs. ~ 80% in the control. The authors suggest that this effect is attributable to the ability of AESIS-1 to stimulate fibroblast migration. Moreover, an increase in various chemokine receptors (CCR1, CCR3, CCR7, CCR9, CXCR1, and CXCR2) necessary in the regulation of cell migration, tissue remodelling, and angiogenesis was observed as well.

Another factor responsible for the delayed repair or non-healing of ulcers is the presence of an infection or of a biofilm in the wound field. Vivcharenko et al. [3] present an overview of infections/biofilms as one of the main complications negatively affecting the healing process. Moreover, they present the state-of-the-art technological approaches for the topical treatment of wounds, such as nanosponges, foams, gels, nanofibers, hydrogels, and films, which employ the most common antibiotic agents and natural compounds for infection treatment.

The choice of the most appropriate treatment for an infection/biofilm depends on the knowledge of both the aetiological agent and of the compounds to which it is susceptible. This is particularly important in case of biofilms, which are often very difficult to treat.

Paleczny et al. [4] proposed an in vitro method to assess treatment efficacy in order to avoid exposing patients to unnecessary procedures.

The authors used a medium simulating the wound milieu to produce an in vitro biofilm model of the strains that represent the main causes of wound infections: *S. aureus*, *S. epidermidis*, *P. aeruginosa*, *C. albicans*, and *E. coli*. Biofilms prepared under standard microbiological culture conditions were used for comparison. The authors observed important differences between the two kinds of biofilms; in fact, the biofilm they developed was more metabolically active, thicker, and more aggregated than the standard one. This suggests that their model is closer to the real-life conditions of a wound bed and that it is thus useful for the screening of therapeutic options in order to identify which is the most suitable.

Wound healing can be promoted by molecules able to interfere with its characteristic processes, such as inflammation. Gushiken et al. [5] formulated the molecule oleoresin (1%) from Copaifera langsdorffii in nanostructured lipid carriers formulated as an emulgel suitable for wound application. They demonstrated their formulation’s ability to promote wound healing via in vivo studies. Oleoresin’s effects were attributed to many activities, including (i) wound retraction mediated by α-smooth muscle actin; (ii) increase in the anti-inflammatory cytokine IL-10 and decrease in pro-inflammatory cytokines; (iii) improved re-epithelialization and tissue remodelling, mainly attributable to collagen synthesis stimulation.

Lipid nanoparticle technology was also used by Angellotti et al. [6] for the topical application of resveratrol. This molecule is able to reduce the inflammatory cascade and the production of free radicals, which responsible for non-physiological conditions that impair the wound repair process. In vitro assays on fibroblasts confirmed the formulation’s cytocompatibility and wound-healing capacity. Moreover, its inhibitory effect on *S. aureus* biofilm formation was demonstrated as well.

The topic of this Special Issue highlights the importance of considering numerous aspects that can contribute to the healing process. This collection features research papers dealing with various strategies/diagnostic tools which can be useful in the treatment of wounds. In order to achieve it, it is crucial to know the processes involved in tissue repair as well as the complications that can delay healing. 

Developing a single treatment applicable to any type of wound is simply not possible. As wound healing is the result of many interconnected processes which vary from patient to patient and from one wound type to another, it is key that clinicians can quickly identify the most suitable therapy in each case through ad hoc studies. 

With this in mind, it is fundamental that a large number of molecules and delivery systems that can meet the numerous needs of patients are available in order to improve the quality of life of the ever-increasing number of people affected by chronic wounds.
